# Screw Fixation as the Primary and Definitive Treatment of an Isolated Uncommon Fracture of the Anterior Margin of the Distal Tibia

**DOI:** 10.7759/cureus.33438

**Published:** 2023-01-06

**Authors:** Stamatios A Papadakis, Dimitrios Pallis, Konstantinos Tsivelekas, Margarita-Michaela Ampadiotaki, Dimitrios Segos

**Affiliations:** 1 Second Department of Orthopaedics, KAT Attica General Hospital, Athens, GRC; 2 Orthopaedic Surgery, Private Practice, Athens, GRC

**Keywords:** tibia plafond, pilon, screw fixation, fracture, tibia

## Abstract

Tibial plafond fractures constitute one of the most challenging fracture types while they are commonly associated with soft tissue damage and severe bone comminution. We present the clinical outcomes of screw fixation as the initial and definitive treatment of an isolated uncommon fracture of the anterior margin of the distal tibia. This is a case of an uncommon type of fracture of the distal tibia. The patient underwent a successful screw fixation and the fracture healed in three months. There was no bone and soft tissue infection. Sixteen months after the injury, an excellent function of the ankle joint was noted. Although fractures of the anterior margin of the distal tibia are uncommon high-energy injuries, uneventful healing with very good functional results can be achieved with screw fixation as the initial and definitive treatment.

## Introduction

Axial-loading fractures of the tibial plafond are one of the most challenging fracture types. The defining character of a tibial pilon fracture is the supraarticular metaphysis involvement that exhibits varying degrees of impaction. This impaction combined with comminution, instability, cartilage damage and joint surface incongruity contributes to the uncertain outcome of the management of this fracture type [1]. The portion of the tibial plafond that sustains the major impact of the talus is determined by the position of the foot at the time of axial loading. Plantar flexion injury results in a posterior lip fragment. Neutral ankle injury results in both posterior and anterior fragments. Dorsiflexion injury results in a fracture of the anterior margin of the tibial plafond [2]. This type could be coded in the 43B generic system according to the AO Foundation/Orthopaedic Trauma Association (AO/OTA) classification system of distal tibial fractures and is considered to lie in the spectrum of severity between simple malleolar fractures and pilon fractures [3,4]. In this case report, we describe a case of an isolated uncommon fracture of the anterior margin of the tibial plafond and its clinical outcome. To our knowledge, there is no other report of the clinical outcome of this uncommon distal tibia fracture managed with screw fixation as the initial and definitive treatment. OTA and the AO Foundation have stated that infrequent fracture patterns have no need to have a unique code, as they could easily be coded by a shortened generic system [3]. However, we believe that this report could be helpful and be considered in a future revised version of the AO/OTA classification.

## Case presentation

**Figure 1:**
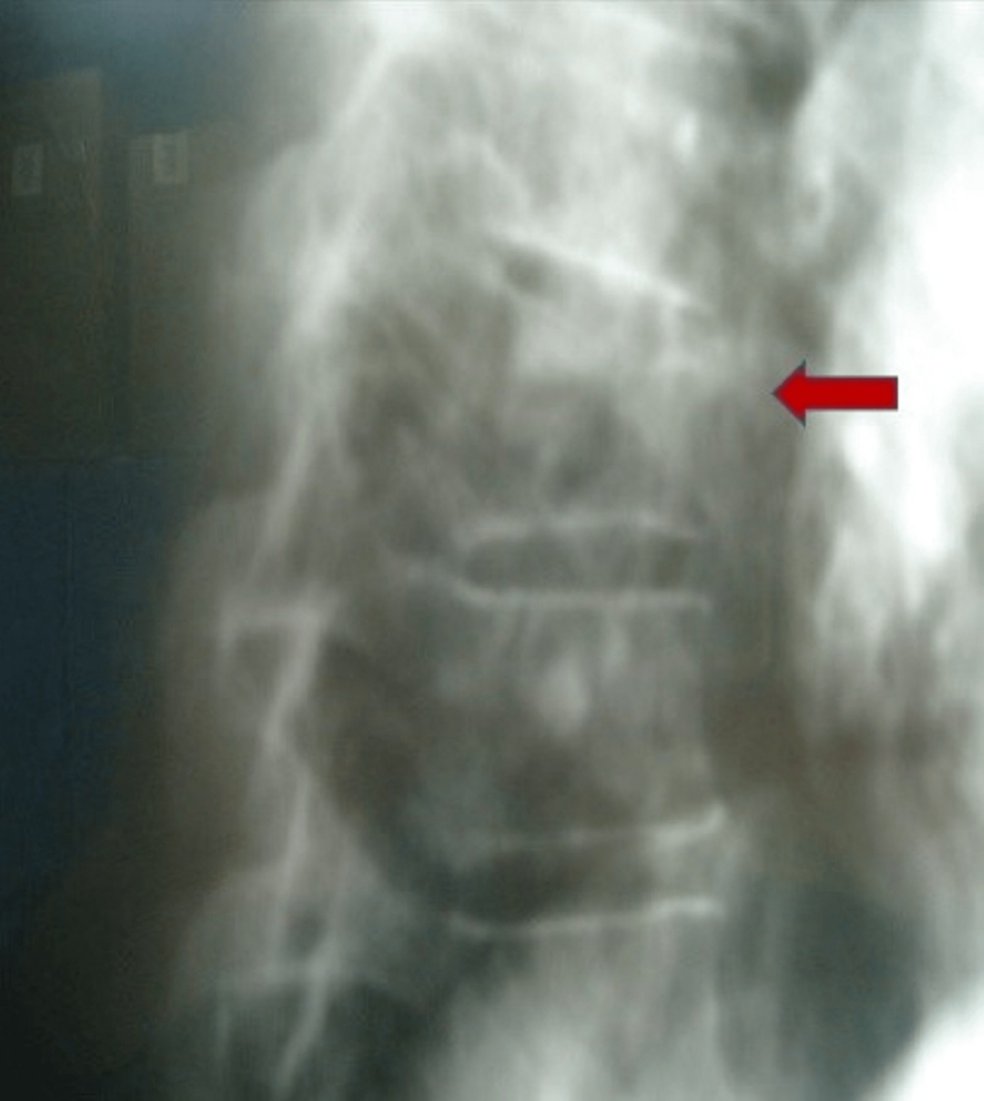
Lateral X-ray of the thoracic spine. The red arrow is the showing the T8 fracture.

**Figure 2:**
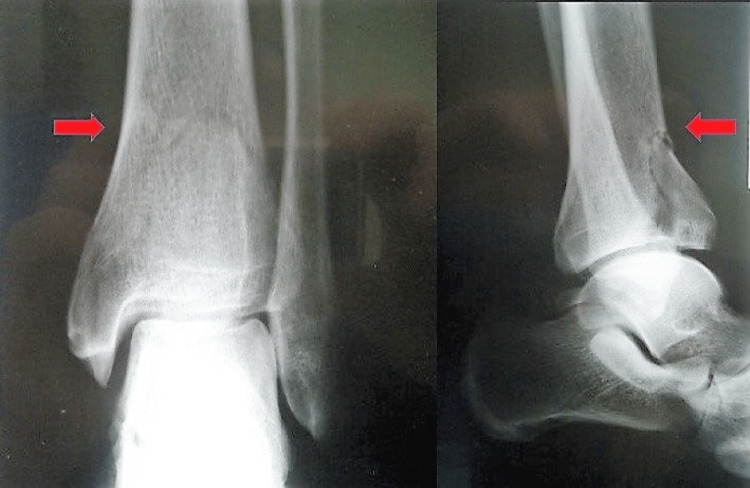
Anteroposterior and lateral X-ray indicating the uncommon type fracture of the anterior margin of the distal tibia (red arrow)

A 50-year-old man fell from a 2-m height onto his right foot. After the fall, the patient indicated that he was unable to stand and was transferred to the emergency department of our hospital. The physical examination revealed a swollen ankle and tenderness of the ankle joint more intense at the anterior surface of the distal tibia. The range of motion of the ankle joint was very painful and hence limited. In addition, the patient complained of pain in the thoracic spine with localized tenderness at the eighth-ninth thoracic vertebrae region. There were no neurological deficits. The history of the injury revealed that during the fall, the patient landed on a rock in a way that the foot was forced to dorsiflexion. The X-ray examination revealed a displaced fracture of the anterior margin of the distal tibia without high comminution and a stable compression fracture of the body of the T8 vertebra (Figures 1, 2).

**Figure 3:**
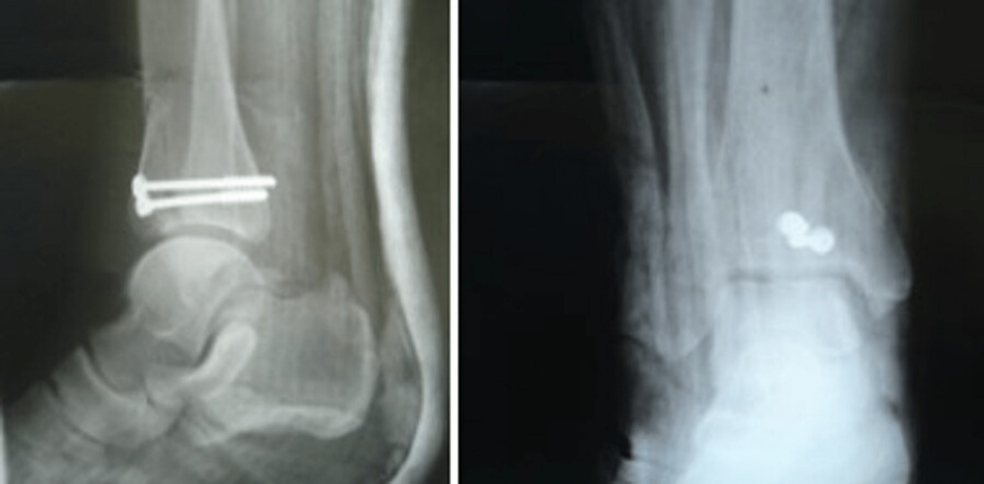
Anteroposterior and lateral postoperative (14 days) X-ray

The patient underwent surgery six hours after his admittance to the emergency department. We exposed the ankle joint anteriorly using a 4-cm longitudinal mid-anterior incision. After incising the superior extensor retinaculum, we retracted the extensor digitorum longus tendon laterally and the anterior tibial vessels and the deep peroneal nerve medially. Reduction of the fracture using the cloth forceps was followed by screw fixation with two cancellous, 3.2-mm lag screws placed anteroposteriorly. We used a washer in each screw. Reduction of the fracture and proper screw placement were confirmed fluoroscopically intraoperatively and roentgenographically postoperatively (Figure 3).

**Figure 4:**
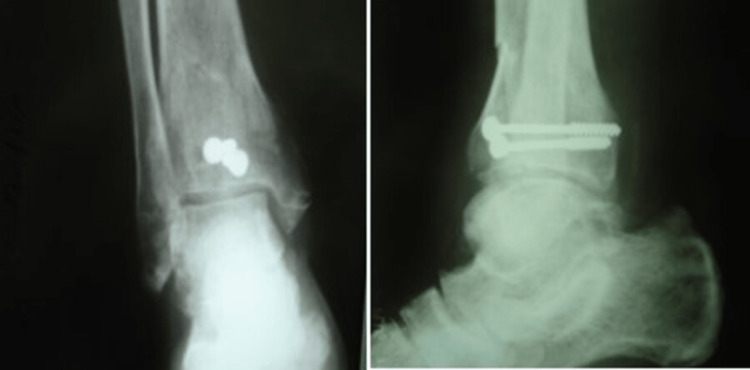
Anteroposterior and lateral postoperative (12 weeks) X-ray

The ankle was immobilized in a neutral position using a posterior caster splint and the patient was given instructions to strictly maintain the limb elevated. The patient was discharged on the third postoperative day without any sign of excessive swelling or vascular compromise. The patient was encouraged to initiate active motion in the splint from the third postoperative day. The splint was removed three weeks after the operation and then a more aggressive range of motion was initiated. Partial weight bearing was allowed in the eighth postoperative week, whereas full weight bearing was allowed after the 12th week. Healing of the fracture was achieved three months after the injury (Figure 4). Six months after the injury, functional results were found to be excellent with a full range of motion and no pain in any activity. Excellent function was still noted at the latest follow-up 18 months after the injury.

The fracture of the T8 vertebra was managed conservatively with a three-point thoracolumbar Taylor brace and healed uneventfully three months after the injury.

## Discussion

Tibial plafond fractures represent approximately 1%-10% of tibial fractures and are commonly associated with soft tissue damage and bone comminution [5]. DiChristina et al. reported two cases of type B3 distal tibia fractures managed with external fixation and internal fixation (screws) [6]. Dickson et al. reported type B3 tibial fractures managed initially with external fixation and subsequently (after two to three weeks) with internal fixation [7]. Hahn and Thies recommended initial external fixation and limited open reduction of the articular surface and subsequent definitive stabilization of this fracture type [8]. Whittle and Wood suggested treatment similar to that for the posterior margin of the tibia but in reverse [4]. Harris et al. reported two cases of type B3 fractures managed with internal fixation only but did not report their clinical outcome [9]. However, the majority of published papers do not differentiate exact types of fractures according to the AO/OTA classification. Group 43B represents tibia, distal end segment, partial articular fracture and is divided into three subgroups (43B3.1, 43B3.2, and 43B3.3) [3]. The fracture characteristics of our case were as follows: it was located anterior, partial articular with comminution at its proximal end with no depression of the articular surface. Thus, it was difficult to categorize it in a specific subgroup. This is in accordance with the AO/OTA statement that the classification is a flexible, evolving system and changes are made based on criticism, users’ feedback, and appropriate clinical research.

Many factors affect the final outcome, including the severity of the fracture, soft tissue damage, and quality of the reduction. Anatomical reduction has not been correlated in some cases with good clinical outcomes [10,11]. The ideal treatment option remains controversial and probably it depends on the severity of bone and soft tissue damage. The common treatment for definitive fixation is the open reduction internal fixation but it compromises the blood supply in the fracture area and increases the rate of infections and soft tissue complications [12]. Although minimal techniques do not allow direct visualization of the fracture area, indirect reduction is achieved with the minimally invasive plate osteosynthesis (MIPO) technique and satisfactory clinical outcomes have been described [13]. External fixation is a damage control treatment option for open fractures or in cases with extensive soft tissue damage. Also, it can be combined with limited percutaneous fixation using lag screws. Fractures without severe comminution and that are easily reducible by external manipulation or traction can be treated with limited internal fixation [14].

According to our findings, the fracture in this case was due to dorsiflexion as the right foot of the patient landed on a rock. This resulted in this uncommon type of fracture that was difficult to classify. It was managed with anteroposterior screw fixation (one or two 3.2-mm cancellous lag screws depending on the size of the fragment) as we believed that this was a suitable treatment option. We considered the external fixation unnecessary, although this type of fracture is a high-energy injury, as screw fixation combined with cast immobilization for three weeks seemed to be stable enough. We believe that the time of surgery played an important role, as endodermal edema, a possible complication in such injuries, was not noticed in this case. Moreover, Mandracchia et al. stated that delayed intervention of distal tibia fractures makes the achievement of the reduction more demanding [15]. A short delay is not unacceptable in less severe cases with minimal or no contusions.

The anatomical reduction of the articular surface of the tibial plafond should be attempted in a way to avoid any compromise of ankle stability or articular surface congruity. We chose the anterior exposure of the ankle joint, as this facilitated the insertion of screws. However, a surgeon might find it difficult to achieve reduction using this approach. Mobilization of the ankle joint should be encouraged as early as possible. Partial weight bearing could be allowed after the eighth postoperative week whereas full weight bearing should not be permitted before the 12th week.

## Conclusions

Fractures of the anterior margin of the distal tibia are uncommon high-energy injuries. We present an isolated uncommon fracture of the anterior margin of the distal tibia that was difficult to classify according to AO/OTA classification. Uneventful healing with very good functional results can be achieved with screw fixation as the initial and definitive treatment. Orthopaedic Trauma Association and the AO Foundation have stated that infrequent fracture patterns have no need to have a unique code, as they could easily be coded by a shortened generic system. However, we believe that this report could be helpful and be considered in a future revised version of the AO/OTA classification.
